# Evidence of a Lunatic Taken in a Case of Manslaughter in a Lunatic Asylum

**Published:** 1851-04-01

**Authors:** 


### EVIDENCE OF A LUNATIC TAKEN IN A CASE OF MANSLAUGHTER IN A LUNATIC ASYLUM.

In the Central Criminal Court, Samuel Hill surrendered to take his trial before Mr. Justice Coleridge and Mr. Justice Cresswell, for the manslaughter of Moses James Barnes.

Mr. Clarkson and Mr. Bodkin conducted the prosecution, instructed by Mr. Undersheriff Law, the solicitor to the Commissioners in Lunacy. The prisoner was defended by Mr. Collier and Mr. Parnell.

Mr. Clarkson, in opening the case to the jury, said that he and his learned friend appeared by direction of her Majesty's Commissioners in Lunacy, for the purpose of laying before them the facts upon which this indictment was founded, and upon which the grave charge of manslaughter was preferred against the prisoner at the bar. An offence of this description was at all times looked upon as most serious, but, in the present instance, the offence was aggravated by the fact, that the unfortunate deceased was a lunatic patient in an asylum, and that the prisoner was a keeper, placed over him, whose duty it undoubtedly was to have treated an unfortunate person in that condition with the utmost kindness and attention. He need hardly observe, that, under these circumstances, the inquiry into which they were about to enter was one of the very greatest public importance, and it appeared to him that the commissioners had not only performed their duty in adopting the present proceeding, but that they would have neglected their duty to the public if they had not done so. The learned counsel then proceeded to state the facts of the case, and he informed the jury that he should be compelled to call one of the patients who was present at the time the fatal attack was made upon the deceased, and who he should, under the sanction of their lordships, offer as a competent witness upon the present occasion.

The following evidence was then adduced:—

William Muncaster deposed that he was an attendant in Mr. Armstrong's asylum at Peckham, and was employed in the infirmary for some time, and the prisoner then took his place. There were generally about seven patients who slept in the infirmary; but when witness left the infirmary, there were about nine or ten, and the prisoner had the charge of them all during the day, and he slept in a room close to them at night. The patients were left at night entirely by themselves. The patients in the infirmary were generally of an inoffensive kind. Witness knew the deceased. He had been a patient in the asylum since March, 1850. He was one of the patients in the infirmary. He died on the 2nd of January. Witness first heard that he had received some injury on the 27th of December. The prisoner gave him the information, and wished him to go and look at the deceased, as he thought he had received some injury. He went to the infirmary, and while the deceased was in the act of changing his shirt, he noticed that his left shoulder looked as though it were broken, or out of joint, and there was a swelling on the shoulder the breadth of a man's hand. The prisoner said he could not imagine how it had happened, and witness remarked that he must have had a severe fall to have caused it, and that it must have been done two or three days. The prisoner then said that the doctor must be made acquainted with it directly, and at that moment Mr. Burton, one of the medical officers, came in. He saw the prisoner often during the three or four preceding days, and he made no communication to him respecting the deceased; and if he had been aware of any injury happening to a patient, it was his duty immediately to have given information to the medical gentleman who resided in the establishment. Witness had seen the deceased on the previous day. It was the duty of the prisoner to dress him, or to see that he was dressed, and it was also his duty to wash him. The deceased was a sullen, obstinate person.

Cross-examined.—The infirmary was upstairs, and the prisoner was the sole attendant in it; but witness went there occasionally, and he could see what the patients were about. The prisoner's conduct towards the patients always appeared to be very kind and attentive. Some of the patients slept out of the infirmary, and the prisoner had to take care of them also. The prisoner was assisted by some of the patients to take care of the others. Attwood, Taylor, and Donelly were those who were so employed. He did not know that they washed or dressed the patients, but they assisted in scouring the floors, carrying things down from the bed-rooms, and he had occasionally seen them help to dress the patients, and also help to feed them. The attendant was sometimes obliged to leave the patients, but he did not think he was ever away so long as half an hour. Attwood and Donelly laboured under delusions. Donelly was under the delusion that spirits were continually about him, and, so far as he was aware, that delusion was never absent from him. The delusion was, that he had spirits continually about him; that he had spirits in his ears, and that the spirits had lost their lips and noses. Attwood, the other patient, was very excitable, but he was not aware that he laboured under any particular delusion. Deceased was a gloomy, sullen kind of man, and he had frequently heard him make use of very bad language, not only to himself, but to the other attendants. The prisoner always appeared to have acted with forbearance both towards him and the other patients.

Re-examined.—The patients were usually washed after breakfast, about half-past nine o'clock. The water was fetched either by the attendant or by one of the patients, if the attendant chose to send him. He never upon any occasion saw a patient dressing another patient alone and in the absence of the attendant.

By the Court.—Two or three days before the 27th December he heard the deceased complain of having the sensation of having a board in his stomach, and he wished witness to take it out.

By Mr. Bodkin.—He laboured under the delusion that he had boards in his stomach and back.

Mr. J. T. Burton deposed that he was the medical superintendent to Mr. Armstrong's establishment at Peckham. It was a private asylum. He knew the deceased. He came to the establishment on the 30th March, 1850. Witness first heard he had received some injury on the 27th December. The prisoner reported the fact to him about ten o'clock in the morning. Witness immediately proceeded to the infirmary ward, and the prisoner stripped the deceased, and he immediately observed that the left arm was fractured close under the shoulder-joint, and the joint was very much bruised and inflamed. There was also a mark on the arm as if produced by a grasp. The bruises extended all round the chest. In witness's opinion these appearances were attributable to violence, such as a fall upon the shoulder—not an accidental fall—but a fall occasioned by some other person. Witness ordered deceased to be put to bed, and sent to Mr. Fidler, the resident surgeon. He remarked to the prisoner that it must have occurred two or three days, and the prisoner replied that he had been complaining of having wounds on his shoulder, but he was always talking nonsense of that sort, and he took no notice of it. Witness told him he must have had a violent fall, and the prisoner said he knew nothing about it. The deceased could not have dressed himself on the previous day, on account of the injury. In witness's opinion, at the time he saw the injury, it must have been at least of three days' standing, and he did not think that any person who dressed him could have failed to observe the injury. The deceased could not have raised his arm at all after receiving the injury, and it must have been perfectly useless to him. It would have been the prisoner's duty to report any accident to him immediately.

By Mr. Collier.—Witness went into the infirmary every day himself, and on the 26th December he looked at the deceased's head, but did not observe anything the matter with his shoulder at that time. The deceased laboured under the delusion that his belly was as big as a butt. Witness never had any occasion to complain of the conduct of the prisoner. The deceased never made any complaint to him. The prisoner appeared very anxious about the injury. The memory of the deceased was affected. He was capable of giving a correct answer to a question, but was certainly a lunatic in the ordinary meaning of the term. Witness was aware that Attwood, Donelly, and Taylor were in the habit of assisting the prisoner, and he had seen them arranging the dress of the other patients, but he did not remember having seen them feeding them. Donelly laboured under a delusion, but he was not aware that Attwood or Taylor laboured under any particular delusion. The delusion of Donelly was always present in his mind when his attention was directed to it, and witness had never known him free from that delusion. Attwood was incapable of carrying on a connected conversation, and was, at times, very abusive, and of very irritable temper. Taylor was a man of weak mind, and incapable of taking care of himself; and they were both lunatics, and unfit to go at large. Donelly was decidedly a lunatic in the ordinary sense of the term.

By Mr. Clarkson.—Barnes was able to give a rational account of an ordinary transaction. With respect to Donelly, he laboured under a delusion that he had spirits in his head, but, in his opinion, he was quite capable of giving a rational account of any transaction that passed before his eyes, and he had always found him able to give an account of anything that had happened to him, and he was always rational, except with regard to the delusion of having spirits in his head, and on that account only he considered him a lunatic. In every other respect he looked upon him as a perfectly sane man.

Dr. Hill deposed that he formerly occupied the office filled by Mr. Burton, who succeeded him. Witness was at the asylum on the 27th of December, and found the deceased was suffering from a fractured arm, and, according to his opinion, that injury had been occasioned by a severe fall on the back part of the shoulder, and that it could not have arisen from a mere ordinary fall by the man's own weight. The deceased was rather tall, but a very spare man, and at this time he was in a very feeble state. Witness put some questions to the deceased, and he told him how he had received the injury, and by whom it had been inflicted. The prisoner shortly afterwards came into the room, and witness said to him, "This is a sad occurrence, the man's arm is broken." The prisoner replied, "I assure you I know nothing about it." Witness said, that the deceased said he had done it, and Donelly said that he saw him do it. The prisoner replied that it was false, and he had never lifted his hand against him. Donelly then interfered, and said, "You know you did do it. You took hold of him in this way." Donelly then laid hold of the prisoner as if to show him how it had happened, and the prisoner again declared that what he had stated was false. Witness remarked that the injury must have been inflicted several days before, and the prisoner said he could assure him that he knew nothing of it until that morning. The conversation of the deceased at this time was quite rational, and he seemed to understand perfectly the conversation which passed between him and the prisoner. Witness had had his attention directed to the insane for some years, and his experience had taught him that persons of unsound mind would bear personal injury and pain without complaining.

By Mr. Collier.—The memory of an insane person was not necessarily affected; it frequently was, and it frequently was not. In a great number of cases the memory certainly was affected. Madness was generally accompanied by a great degree of irritability in the brain. In cases of acute madness, the ideas would succeed each other much more rapidly than in a sane subject. A man might labour under a particular delusion without that delusion affecting his mind particularly upon other subjects; but in most cases where such a delusion existed, the mind was certainly to some degree affected upon other subjects. The only delusion that Barnes appeared to labour under was, that his stomach was as full as a butt, and could not contain any more, and this was the chief reason why he refused to take food. It was difficult, in some cases, to ascertain the extent of a madman's delusion; and they frequently succeeded in concealing their delusions from a medical man, particularly when they become aware that those delusions were the ground for their detention. He had known cases where a madman had pretended that he no longer laboured under a particular delusion, in order that he might obtain his liberty. It was also common for certain classes of madmen to exhibit a great deal of dissimulation. Before Barnes was admitted to the asylum he had refused to take food, and it was necessary to feed him all the time that he was in the establishment. Donelly was, in his opinion, in the strict sense of the word, a lunatic. He had a peculiarity of manner, but he was not excitable. Witness had questioned him repeatedly upon the subject of his delusions about spirits, and he always found him labouring under the same delusion. When witness asked the deceased who had hurt his arm, he replied, "The keeper;" and when he asked him which keeper, he said he did not know, or he could not recollect his name. He then mentioned the names of two keepers, and deceased said they were not the men; and he then asked him if it was Hill, and he replied that it was.

By Mr. Bodkin—The deceased, from his obstinacy and other circumstances, was undoubtedly a troublesome patient.

Mr. A. Poland deposed that he was assistant-surgeon at Guy's Hospital, and demonstrator of anatomy at that institution. On the 3rd of January last he made a post-mortem examination of the body of the deceased. The witness described the nature and extent of the injuries the deceased had received. There were extensive bruises on both sides of the body, the left arm was fractured, and the lower portion of the bone driven completely up into the arm-pit. The sixth and seventh ribs on the right side, directly opposite, were also broken, as well as the eleventh and twelfth ribs, and the broken portions had penetrated the lining membrane of the chest, and had produced extensive inflammation. The head presented the ordinary appearance in the post mortem examination of lunatics. The substance of the skull was thickened, and the brain partly absorbed. In witness's opinion the injuries had been inflicted between the period of four to ten days preceding, and in his opinion they were all sustained at the same time, and that they were the result of great and inordinate violence, and could not have been occasioned by a simple fall, or by the man himself. The injuries he considered might have been occasioned by the deceased being tripped up and thrown to the ground, but it appeared to him that when he so fell the person who had thrown him must have fallen on him, and this would be the most likely mode for such injuries to have been received. The injuries the deceased had received were undoubtedly the cause of death; but the immediate cause of death was exhaustion consequent upon these injuries.

By Mr. Parnell—The head of the deceased presented all the indications of his being a confirmed lunatic. The lungs of the deceased were diseased from long standing consumption. The double character of the injuries led him to believe that the deceased could not have occasioned them himself by a fall.

Mr. J. S. Flower, another surgeon who assisted on the *post mortem* examination, confirmed the testimony of Mr. Poland with regard to the nature of the injuries received by the deceased, and the cause of death.

By Mr. Collier—There was a great number of separate bruises upon the deceased's body, which had nothing to do with the broken arm, and they might, in his opinion, all have been the result of severe beating, either by the fists, or by some blunt instrument, and whoever injured the deceased must have exercised very great violence; and the injuries did not appear to him to be such as would be the result of any ordinary conflict.

By the Court—The injuries might have been occasioned by one heavy man falling upon another.

By Mr. Collier—The whole of the injuries might certainly have been produced by one heavy fall.

By Mr. Clarkson—If the deceased had been lifted up from a bed and thrown with great violence upon the ground, and afterwards knelt upon, it would account for the whole of the appearances observed upon the body of the deceased.

Mr. Bodkin said that he now proposed to call Richard Donelly, the lunatic patient, as a witness.

Mr. Collier said he should submit to the court that enough appeared upon their lordships' notes to make it quite clear that he was not an admissible witness, as being a lunatic.

Mr. Justice Coleridge said that unless the learned counsel could cite any case in which it had been ruled that a lunatic of the character of this person was not a competent witness, the court should certainly receive his testimony, and reserve the point for further consideration, if such a course should become necessary. He believed the question had never been decided.

Mr. Collier admitted that he was unable to cite any decision, but he apprehended that it was contrary to every principle of the English law that a lunatic should be permitted to give evidence.

The court said that they should allow the witness to be examined if it should appear that he was aware of the nature of the obligations of an oath, and upon that point they would allow the learned counsel an opportunity to examine him upon the voir dire.

Richard Donelly, the person referred to, was then brought into court, and he was examined by Mr. Collier.

In answer to the questions that were put to him, he said he was aware that he had a spirit; he said that he had twenty thousand spirits. They were not all his own spirits, and he did not know whose they were, but he would inquire. His own spirits he said he could recognise as being those which ascended from his stomach to his head, and those which were in his ears. He considered that these spirits were created by the palpitation of the nerves.

Mr. Collier asked him whether these spirits ever spoke to him?

He replied that they did incessantly, and particularly at night.

In answer to further questions of the same kind, he said he believed that these spirits were immortal, and that they would live after he was in the grave.

Mr. Collier inquired if he was aware where these spirits came from?

Donelly said that he believed they came from various disorders and from various people. He believed that some came from the Queen, for she was in the habit of constantly visiting him. He also said that Luther and Calvin, and "all those controversial spirits," occasionally came to visit him, but, he said, there was goodness in them. These spirits were often speaking to him, and they were speaking to him now He was not himself a spirit, but flesh and blood, and when his body went to the grave, his spirit would survive him.

Mr. Collier—Where do you expect your spirit will go when you are dead?

Donelly—I cannot say; perhaps to heaven, or perhaps to purgatory.

Mr. Justice Coleridge—Do you believe in purgatory?

Donelly—I do. I am a Roman-catholic, and I have been brought up in the fear of purgatory from my infancy.

By Mr. Bodkin—I understand the meaning of taking an oath. I have been taught by my Catechism that it is lawful to swear for God's honour and my neighbour's good.

Mr. Bodkin—What does a man do when he swears?

Donelly—I consider an oath is an obligation imposed upon men for the good of the law.

Mr. Bodkin—Do you appeal to anybody when you take an oath?

Donelly—Certainly, I appeal to the Almighty, and I believe that if a man takes a false oath he will go to hell to all eternity.

Mr. Collier was then about to re-examine the witness, but Mr. Clarkson contended that he had no right to do so, and he, at the same time, complained that the learned counsel had not put a single question to the witness applying to the point whether he understood the nature of an oath, but that all his questions related to subjects calculated to excite the witness.

Mr. Collier denied that he had any such object, and said it appeared to him that all the inquiries he had made tended to ascertain whether the witness really understood the sacred obligation of an oath.

The Court then ruled that the witness should be examined.

Donelly was accordingly sworn. He said — I am an Irishman. I have been in the establishment at Peckham four years and four months exactly, yesterday. I went on the 14th October. I used to be in the infirmary occasionally. I knew the deceased man Barnes, and I used to attend upon him. Taylor and Attwood were two other patients in the infirmary. I know the prisoner. He was one of the keepers. I remember a little time before Christmas-day, at bedtime, that the deceased would not go to bed. He did not like going to bed, and I told Hill (the prisoner) on that night that he would not go to bed. The prisoner went up to him as he was sitting on the bed, and laid hold of him to put him to bed, and he threw him rashly on the floor, and they both went down together, and the patient was 'hurted.' I know that he was hurt by the report of the doctor, and my own observation that his hand was swelled. They both got up together, and Barnes was then put to bed, and I said to him shortly afterwards, 'You have got your Christmas-box.' Barnes complained to me after this that he was hurt, and I examined him and thought his collar-bone was broken. I saw Hill the next morning when the patients were washed and dressed, but I am not sure that Barnes complained at that time. I believe that I dressed Barnes after this in Hill's presence, and that he heard him complain of pain in his arm, and upon one occasion Hill lifted the arm up, and when he left go of it the arm fell down, as though it was dead or powerless.

By Mr. Collier. — The deceased objected to the other patients undressing or dressing him, and we frequently disagreed about it. The prisoner, however, used to put him to bed whether he liked it or not. Sometimes Barnes would let them dress him, and sometimes he would not. Attwood and Taylor used to assist in dressing and undressing him. Attwood was a man whose passion was very easily raised, and I have frequently seen him very angry with the deceased, and once he pushed him down upon a form, and the prisoner interfered and checked him. I believe that upon another occasion he laid hold of Barnes and struck him. Taylor is also a very passionate man, and the only way to keep him quiet is to give him tobacco. He is apt to be very violent if you don't look after him, but yet he assists both the keepers. I myself thought the occurrence took place on the Monday before Christmas-day, but the spirits want to make me believe that it was Tuesday.

Mr. Justice Coleridge to the witness—Is the account you have just given us of the transaction, an account of what you yourself saw, or is it what these spirits have told you took place?

Donelly—My lord, I have only told you what I myself was an eye-witness of. The spirits only want to make me believe that I am mistaken in the day, and that it was Tuesday instead of Monday, but I myself believe it was Monday. (The occurrence in reality did take place on the Monday.)

Mr. Clarkson said this was the case for the prosecution.

Mr. Collier then addressed the jury for the defence, and after remarking upon the unusual nature of the charge, and the extraordinary character of the evidence by which it was sought to be supported, he said it was perfectly clear that the only evidence which in any way identified the prisoner as the person who had committed the violence, was that of the witness Donelly, and he urged upon them the danger that would result from convicting a person of so serious a charge as this, or indeed of any charge whatever, upon the evidence of a person who was admitted to be a lunatic. It had been ingeniously argued that the prisoner was perfectly rational upon every subject but that of the particular delusion under which he laboured; but he asked the jury whether it could be safe to place any reliance upon the evidence of an individual whose mind, it was admitted, was diseased. How was it possible for the jury to distinguish how far the delusion in his mind extended, or where sanity ended and insanity began; and for all that appeared to the contrary, the whole of the statement he had made might have been a delusion. He did not, in making these observations, at all intend to complain of the inquiry having been instituted; on the contrary, he thought it a most proper one, and he agreed with Mr. Clarkson, that the commissioners would not have performed their duty if they had not instituted a strict inquiry into all the circumstances, where the death of an unhappy person of this description was involved. The learned counsel then called the attention of the jury to the medical testimony, and to the extent of the injuries received by the deceased, and he argued that it was more probable that those injuries should have been occasioned by a desperate struggle between a number of madmen left together during the night without an attendant, than that they should have been wantonly inflicted by the prisoner, who had always borne the character of a kind humane man. He concluded by observing that the present inquiry, he had no doubt, would at all events have this one good effect, of doing away with the practice of leaving a number of madmen thus without proper restraint, a practice which he could not help saying appeared to him most improper, and which had most probably led to the present unhappy occurrence.

Mr. Justice Coleridge having summed up the whole case,

The jury, after deliberating a short time in the box, retired. After being absent about half an hour, they returned into court and gave a verdict of *Guilty*, but at the same time strongly recommended the prisoner to mercy on account of his previous good character.

Mr. Justice Coleridge said that judgment would be postponed, in order that the opinion of the judges might be taken as to the admissibility of the evidence of the witness Donelly.

